# The Sr-HA-loaded PLGA cage structure combines cells to construct a bone tissue repair unit

**DOI:** 10.1093/rb/rbag051

**Published:** 2026-05-05

**Authors:** Huixing Yi, Guowen Duan, Siyu Li, Dongbiao Chang, Lulu Han, Yinhao Lai, Jun Sheng, Suiyan Li, Jie Weng

**Affiliations:** School of Life Science and Engineering, Southwest Jiaotong University, Chengdu, Sichuan 610031, China; School of Life Science and Engineering, Southwest Jiaotong University, Chengdu, Sichuan 610031, China; Key Laboratory of Advanced Technologies of Materials Ministry of Education, School of Materials Science and Engineering, Southwest Jiaotong University, Chengdu, Sichuan 610031, China; Key Laboratory of Advanced Technologies of Materials Ministry of Education, School of Materials Science and Engineering, Southwest Jiaotong University, Chengdu, Sichuan 610031, China; College of Medicine (Institute of Biomedical Engineering), Southwest Jiaotong University, Chengdu, Sichuan 610031, China; Key Laboratory of Advanced Technologies of Materials Ministry of Education, School of Materials Science and Engineering, Southwest Jiaotong University, Chengdu, Sichuan 610031, China; College of Medicine (Institute of Biomedical Engineering), Southwest Jiaotong University, Chengdu, Sichuan 610031, China; College of Medicine (Institute of Biomedical Engineering), Southwest Jiaotong University, Chengdu, Sichuan 610031, China; Key Laboratory of Advanced Technologies of Materials Ministry of Education, School of Materials Science and Engineering, Southwest Jiaotong University, Chengdu, Sichuan 610031, China; Department of Orthopedic, The General Hospital of Western Theater Command of PLA, Chengdu, Sichuan 610083, China; School of Life Science and Engineering, Southwest Jiaotong University, Chengdu, Sichuan 610031, China; College of Medicine (Institute of Biomedical Engineering), Southwest Jiaotong University, Chengdu, Sichuan 610031, China; Key Laboratory of Advanced Technologies of Materials Ministry of Education, School of Materials Science and Engineering, Southwest Jiaotong University, Chengdu, Sichuan 610031, China

**Keywords:** strontium-doped hydroxyapatite, cage-structured PLGA microspheres, cellular microcarrier, bone tissue engineering

## Abstract

Osteoporosis arises from an imbalance where bone resorption outpaces formation, reducing bone mass and mineral density while elevating fracture risk. Subsequent surgical repair of these fractures in compromised bone is prone to cause secondary injury, further increasing the challenges of recovery. In this study, we developed an injectable bone repair unit based on multicellular delivery microspheres, designed to promote the repair of osteoporotic bone defects through the synergistic effect of cells and metal ions. First, cage-structured PLGA microspheres (PLGA-CAS) were fabricated via the double-emulsion technique. Subsequently, the surfaces of the PLGA-CAS were uniformly coated with needle-like strontium-doped hydroxyapatite (Sr-HA) particles synthesized by wet-chemical precipitation to form Sr-HA-coated PLGA-CAS (SrHP). Finally, mouse pre-osteoblasts (MC3T3-E1) and human umbilical vein endothelial cells (HUVECs) were separately cultured on the SrHP microspheres, which together constituted the final bone repair unit. Structural and compositional analyses revealed that the PLGA-CAS scaffold possessed an interconnected porous structure with successfully anchored Sr-HA particles. Both SrHP loaded with MC3T3-E1 (M-SrHP) and with HUVECs (H-SrHP) exhibited high cell viability and injectability. Notably, the blended microsphere system incorporating both M-SrHP and H-SrHP (MH-SrHP) simultaneously facilitated osteogenic differentiation, promoted angiogenesis and inhibited osteoclast differentiation *in vitro*. This multifunctional construct offers a streamlined platform for osteoporotic bone repair and represents a promising therapeutic strategy for clinical translation.

## Graphical Abstract

**Graphical Abstract:**
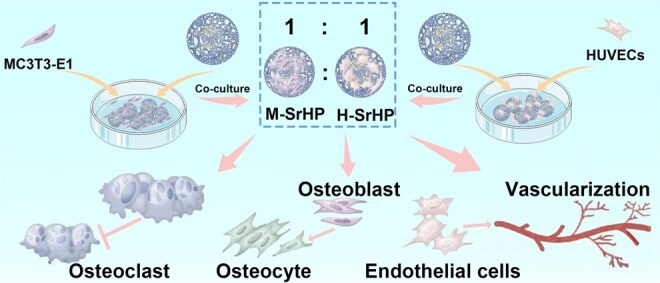

## Introduction

Osteoporosis, a prevalent age-related skeletal disorder, is characterized by an imbalanced bone microenvironment where osteoblast-mediated bone formation declines while osteoclast-mediated bone resorption becomes excessive [1, 2]. When the bone microenvironment in osteoporotic patients is disrupted, the compromised self-healing capacity of the body often fails to restore bone integrity, leading to increased bone fragility and elevated fracture risk [3–5]. Current clinical management of osteoporotic bone defects primarily relies on pharmacological interventions, including nutritional supplements, anti-resorptive agents and anabolic agents [6]. However, systemic drug administration frequently yields suboptimal therapeutic outcomes due to inadequate local bioavailability [7–9]. Current single-mechanism osteoporosis drugs primarily target bone formation or resorption. However, prolonged administration is associated with plateaued efficacy and potential safety concerns, such as atypical fractures or osteosarcoma [10]. More critically, these systemic interventions typically focus solely on regulating bone metabolism, largely overlooking the pivotal role of angiogenesis. Angiogenesis is indispensable for bone defect repair, as it orchestrates nutrient supply and maintains the coupled environment required for osteogenesis. Therefore, the development of innovative therapies with enhanced efficacy and reduced side effects is urgent for osteoporotic fractures, which is crucial for promoting postoperative bone healing [11, 12].

Injectable microspheres present a promising alternative for repairing osteoporotic bone defects. Their injectability enables direct filling of irregular defects with minimal surgical trauma, offering significant clinical potential for personalized minimally invasive therapy [13, 14]. Current constituents for bone repair material fabrication primarily comprise bioactive inorganics, natural polymers, synthetic polymers and composites [15–17]. Poly (lactic‐co‐glycolic acid) (PLGA) is an FDA-approved biodegradable synthetic copolymer valued for its excellent biocompatibility, tunable mechanical properties and controlled degradation kinetics [18–20], which makes it highly suitable for implantable systems to deliver drugs, cytokines and cells [21]. However, its applications in bone regeneration are constrained by inherent limitations such as surface hydrophobicity, acidic degradation byproducts and insufficient osteoinductivity, which can be addressed by incorporating hydroxyapatite [22–25].

Hydroxyapatite (HA) with PLGA offers multiple synergistic advantages [26–28]. As a principal inorganic constituent of human bone, HA demonstrates superior biocompatibility and osteo-inductive potential [29]. The modification of PLGA microspheres with HA imparts a hydrophilic character to the surface, creating a favorable environment for cell adhesion [30–33]. Materials with hydrophilic surfaces not only enhance cellular adhesion but also facilitate the adsorption of extracellular matrix (ECM) components and serum proteins, thereby modulating cellular differentiation and proliferation to promote tissue regeneration and repair [34–36]. However, in the treatment of osteoporotic bone defects, pure HA therapy remains insufficient and limited. Research has confirmed that by leveraging the specific biological roles of different metal ions, doped hydroxyapatite significantly broadens the scope of potential bone repair applications [37, 38]. Notably, due to the dual functions of strontium (Sr) ions in inhibiting osteoclastic resorption and promoting osteogenic differentiation, strontium-doped hydroxyapatite (Sr-HA), thus, holds broad application prospects in the field of bone repair [39–41].

Cell therapy rebuilds bone remodeling homeostasis via osteogenic cell transplantation in osteoporotic models, offering a promising strategy for primary osteoporosis [42, 43]. However, restoring bone integrity requires more than just osteogenesis; it inherently relies on the concurrent re-establishment of a functional vascular supply. This process relies on coordinated interactions between osteogenic cells, endothelial cells and other cellular components that drive coupled osteogenesis and angiogenesis [44–46]. Consequently, multicellular loading in bone tissue engineering constructs is critical for enhancing osteogenic efficacy and bone regeneration outcomes [47–49]. Building upon the well-documented osteogenic potential of mouse pre-osteoblast (MC3T3-E1) implantation for bone repair, strategic co-loading of human umbilical vein endothelial cells (HUVECs) further enhances therapeutic outcomes by conferring pro-angiogenic capabilities to the construct. Research suggests that combining osteoblasts with angiogenic cells has broad application potential in tissue repair, with a 1:1 ratio demonstrating the highest proliferation rate and osteogenic potential [45, 47]. This synergistic approach promotes vascularized bone regeneration through coupled osteogenesis-angiogenesis, which is particularly critical for repairing osteoporotic bone defects [50–52].

**Figure 1:**
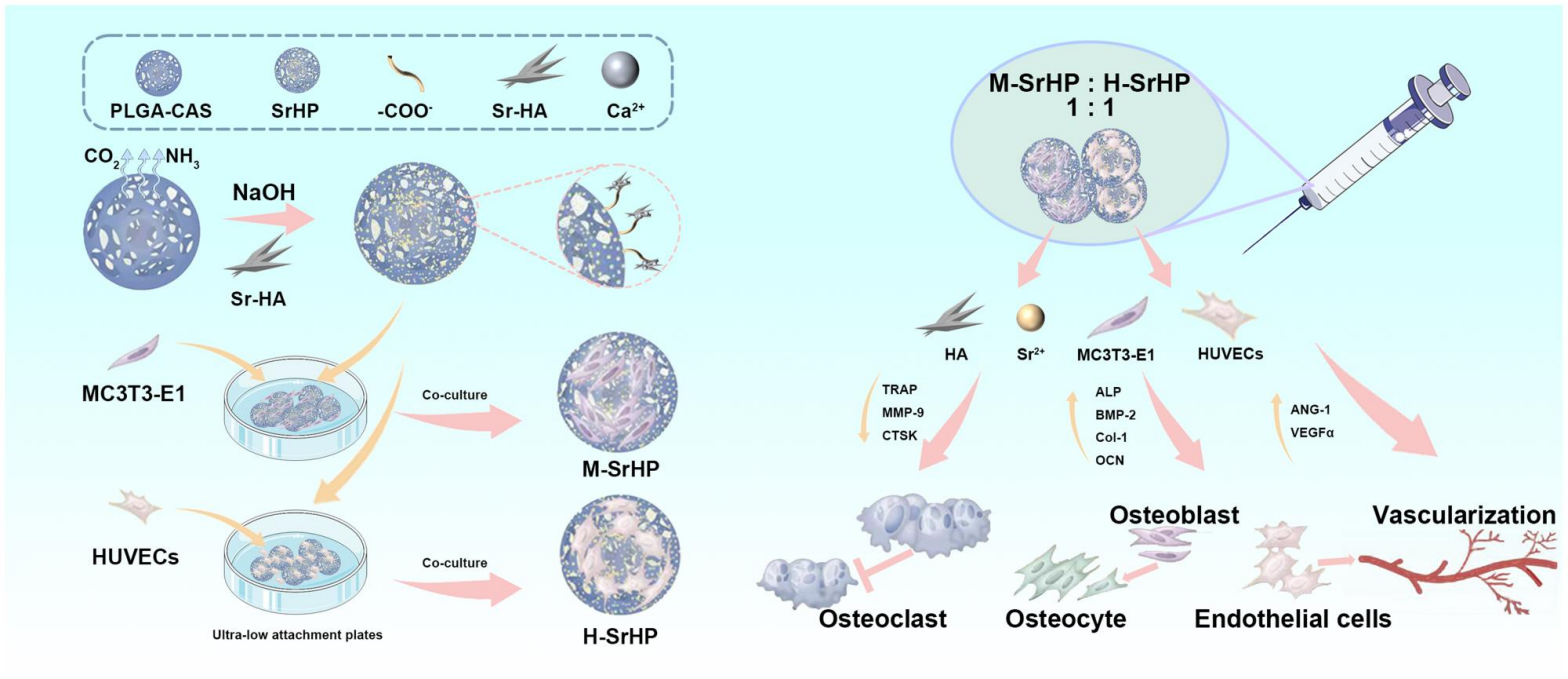
Scheme of the preparation of bone tissue repair units and their regulation of cell behavior *in vitro*. The left panel shows the stepwise synthesis involving PLGA-CAS microspheres, Sr-HA, and subsequent co-culture with MC3T3-E1 or HUVECs to form M-SrHP and H-SrHP. The right panel displays a syringe injecting a 1:1 mixture of these units, with arrows indicating downstream cellular regulation: inhibiting osteoclasts while promoting osteoblast differentiation, osteocyte formation, and vascularization.

This study develops and evaluates an Sr-doped HA/PLGA (SrHP) microsphere system designed as a cellular vehicle to promote bone regeneration in osteoporotic defects. Cage-structured PLGA microspheres (PLGA-CAS) were fabricated via a double-emulsion technique. Sr-HA nanoparticles were synthesized through a wet-chemical process and subsequently adsorbed onto the PLGA microsphere surfaces. Comprehensive material characterization confirmed the successful loading of Sr-HA, which significantly enhanced the hydrophilicity and bioactivity of the composite (Figure 1). Assessments were performed after the fabrication of the composite constructs, in which SrHP microcarriers were co-loaded with MC3T3-E1 cells (M-SrHP) and HUVECs (H-SrHP) to serve as a bone tissue repair unit. These biohybrid systems were rigorously evaluated for cytotoxicity profiles, proliferative kinetics and functional integration within biomimetic microenvironments. The blended bone tissue repair unit incorporating both M-SrHP and H-SrHP (MH-SrHP) unifies cell viability maintenance, osteogenesis promotion, angiogenesis promotion and osteoclast suppression in a single platform for multifunctional bone regeneration.

## Materials and methods

### Materials

Calcium nitrate tetrahydrate (Ca(NO_3_)_2_·4H_2_O), diammonium hydrogen phosphate ((NH_4_)_2_HPO_4_), ammonia (NH_3_·H_2_O), polyvinyl alcohol 1788 (PVA, Mw = 83 kDa), sodium hydroxide (NaOH), strontium nitrate, CHCl_3_, C_3_H_8_O and anhydrous ethanol were purchased from Chengdu Chron Chemicals Co., Ltd. PLGA (LA/GA = 50:50; Mw = 30 kDa) was supplied by Jinan Daigang Co., Ltd. Polyethylene glycol-400 (PEG-400), dichloromethane (CH_2_Cl_2_, DCM) and ammonium bicarbonate (NH_4_HCO_3_) were provided by Tianjin Zhi Yuan Reagent Co., Ltd. Penicillin-streptomycin-amphotericin B solution (100×), trypsin 0.25% (1×) solution and Fetal bovine serum (FBS) were purchased from Cytiva. Dulbecco’s modified Eagle’s medium/high glucose (HG-DMEM) and minimum essential medium alpha basic (1×; α-MEM) were provided by Gibco. A vacuum blood collection tube was provided by Jiangsu Yuli Medical Instrument Co., Ltd. Tartrate-resistant acid phosphatase staining solution (TRAP) was purchased from Wuhan Servicebio Technology Co., Ltd. MC3T3-E1, RAW 264.7 and HUVECs were purchased from Wuhan Pricella Biotechnology Co., Ltd. The Calcein-AM/PI live/dead viability/cytotoxicity assay kit, BCIP/NBT Alkaline phosphatase (ALP) color development kit, 4% Paraformaldehyde Fix Solution (PFA), tetramethylrhodamine-phalloidin (TRITC Phalloidin, 1:200), 4′,6-diamidino-2-phenylindole (DAPI) staining solution, Cell Counting Kit-8 (CCK-8) and 2% Alizarin Red S staining solution (ARS, pH = 4.2) were purchased from Beyotime. Dimethyl sulfoxide (DMSO) was supplied by Sigma. Trizol was purchased from Thermo Fisher. RANKL was supplied by R&D Systems.

### Preparation of SrHP

#### Preparation of Sr-HA

According to a previously established method, synthesis of strontium-doped nano-hydroxyapatite *via* wet-chemical precipitation [38]. Stoichiometric amounts of Sr(NO_3_)_2_ and Ca (NO_3_)_2_·4H_2_O were dissolved in reverse osmosis (RO) water to formulate a precursor solution with a Sr^2+^/Ca^2+^ molar ratio of 0.05 with a final solution concentration of 0.5 M. At 45°C, 100 mL of the Sr/Ca solution was magnetically stirred continuously. In parallel, 100 mL of 0.3 M (NH_4_)_2_HPO_4_ was prepared and adjusted to pH 10.5 *via* dropwise addition of concentrated NH_3_·H_2_O. The phosphate solution was delivered into the Ca^2+^ solution at 5 mL/min using a vertical injection pump (RSP01-BG2, Biotaor, China). Following complete reagent addition, 6 g of polyethylene glycol 400 (PEG-400) was incorporated as a colloidal stabilizer and the reaction system was maintained under continuous stirring at 45°C for 1 h. The resultant suspension underwent static aging for 24 h at room temperature to enable crystalline maturation.

The precipitated Sr-HA particles were purified through centrifugation cycles at 1000 rpm for 10 min per cycle using a high-speed centrifuge (H3-18K, Ke Cheng, China), with alternating washes employing RO water and anhydrous ethanol in a three-round repetitive sequence. Final products were stored in anhydrous ethanol to inhibit particle agglomeration.

#### Fabrication of PLGA-CAS

The PLGA-CAS was synthesized *via* a water-in-oil-in-water (W/O/W) double-emulsion technique. For the oil phase, 200 mg of PLGA was dissolved in 8 mL of CH_2_Cl_2_, followed by the addition of 2.5 mL of 1.5% (w/v) NH_4_HCO_3_ solution. The mixture was emulsified using a high-speed homogenizer (FSH-2A, Jtliangyou, China) at 3600 rpm for 2 min to generate the primary emulsion. Subsequently, the primary emulsion was rapidly transferred into the preheated polyvinyl alcohol (PVA) solution under continuous mechanical stirring at 300 rpm for 2 h to form the double emulsion. After being cleaned thoroughly with RO water, the PLGA-CAS was hydrolyzed with 10 mL of 0.1 mol/L NaOH aqueous solution for 3 min and washed three times with RO water. Finally, the purified microspheres were lyophilized using a freeze-dryer for 12 h to obtain dry PLGA-CAS.

#### Fabrication of SrHP

The ethanol-suspended Sr-HA nanoparticle dispersion was standardized to 10 mg/mL through volumetric dilution. Precisely 20 mg of lyophilized PLGA-CAS was combined with 60 mL of the Sr-HA suspension in a conical centrifuge tube, followed by continuous orbital mixing for 45 min in a thermoregulated shaker (SHZ-82A, Aohua, China) to ensure homogeneous nanoparticle deposition onto the microcarriers. Following the adsorption process, the SrHP suspension was retrieved and subjected to freeze-drying in a lyophilizer for 12 h to yield SrHP.

#### Characterization of SrHP

Bright-field images of Sr-HA/PLGA were captured using an optical microscope (Olympus IX51, Japan). The surface morphology of Sr-HA, PLGA-CAS and Sr-HA/PLGA was visualized using a scanning electron microscope (SEM, JSM-7800F, JEOL, Japan). The surface elements of Sr-HA and PLGA-CAS were determined using X-ray energy dispersive spectroscopy (EDS, OXFORD X-Max 80, Bruker, USA). The crystal structure of Sr-HA was analyzed by transmission electron microscopy (TEM, JEOL, JEM 2100F, Japan).

To detect surface carboxyl groups on Sr-HA, Sr-HA/PLGA and PLGA-CAS, 5‑aminofluorescein was conjugated via an amidation reaction between the carboxyl groups on the materials and the amino groups of the fluorophore, using EDC·HCl and NHS as coupling agents. The distribution of carboxyl groups was then visualized under an inverted fluorescence microscope (Olympus IX51, Japan). The surface elemental composition of Sr-HA, Sr-HA/PLGA and PLGA-CAS was analyzed using an imaging X-ray photoelectron spectrometer (XPS, Axis Ultra, Japan).

The chemical composition of Sr-HA, Sr-HA/PLGA and PLGA-CAS was analyzed using Fourier transform infrared spectroscopy (FTIR, Nicolet 5700, Thermo Fisher Scientific, USA). The phase structures were examined using X-ray diffraction (XRD, Philips PW3040/60, Malvern Panalytical, UK). The thermal stability and composition of the samples were assessed using thermogravimetric analysis (TGA, TGA/DSC3+, Mettler-Toledo, Switzerland).

Membranes were formed using PLGA-CAS or Sr-HA/PLGA, and the water contact angle (DSA100, Krüss, Germany) was determined to assess their wetting properties. For the degradation analysis, 200 mg of each group of microspheres were immersed in a tube containing 10 mL of PBS at pH 7.4 or pH 6.4 and continuously shaken at 37°C for 14 days. The concentrations of metal ions were measured by inductively coupled plasma-optical emission spectrometry (ICP-OES, Agilent 5110). The diameter was analyzed using a particle size analyzer (Multisizer-4e, Beckman Coulter, USA), while pore size was quantified *via* ImageJ software.

The quasi-static compression tests were performed using a universal material testing machine (Instron 5969, USA) at room temperature with a loading rate of 0.5 mm/min.

### Biocompatibility of SrHP

Biocompatibility assessment of SrHP included hemolytic testing at varying concentrations using rat tail vein blood and proliferation assessment *via* CCK-8 assays. To ensure aseptic conditions, the microspheres were sterilized under ultraviolet light for 12 h prior to testing. MC3T3-E1 were cultured under standard conditions (37°C, 5% CO_2_) and seeded into 96-well plates at 5 × 10³ cells per well. After 24 h of cellular adhesion, the culture medium was replaced with 200 μL of sterile material suspension, prepared by blending SrHP with α-MEM supplemented; the concentration of SrHP is 0.5 mg/mL. A negative control group (excluding cells and materials) and a positive control group (including cells but without materials) were also established. After 48 h of co-culture, the supernatant was aspirated and replaced with 200 μL of fresh α-MEM containing 10% (v/v) CCK-8 reagent. After 2 h of incubation, absorbance was measured at 450 nm using a microplate reader (SPECTRO star Nano, BMG LABTECH, Germany). Relative cell viability (%) was calculated as:


(1) $$\mathrm{Relative}\;\mathrm{cell}\;\mathrm{viability}\;(\%)=\;\frac{{\mathrm{OD}}_{\mathrm{sample}}-{\mathrm{OD}}_{\mathrm{negative}}}{{\mathrm{OD}}_{\mathrm{positive}}-{\mathrm{OD}}_{\mathrm{negative}}}\times 100\%$$

Sterilized SrHP were formulated as 0.5 mg/mL suspensions in α-MEM supplemented with 10% FBS and 1% penicillin-streptomycin-amphotericin B solution (100×). MC3T3-E1 was co-cultured with the SrHP suspensions for 1, 3 and 5 days to assess proliferation kinetics. Cellular metabolic activity was quantified using the CCK-8 assay, with absorbance measurements (450 nm) serving as a proxy for viable cell density. Following PBS rinsing, cells were incubated with 100 μL of Calcein-AM/PI Double Stain Kit solution for 30 min at 37°C under light-protected conditions after co-culturing for 3 days. Viability was visualized *via* fluorescence microscopy (Olympus IX51, Japan), where live cells fluoresced green (Calcein-AM) and dead cells red (PI). ImageJ (NIH, USA) was employed for quantitative analysis of fluorescence intensity ratios.

For HUVECs, substituting α-MEM with HG-DMEM supplemented with 10% FBS and 1% penicillin-streptomycin-amphotericin B solution (100×).

### Cell-laden SrHP

To achieve cellular adhesion onto SrHP, a co-culture strategy was implemented using ultra-low attachment 6-well plates (Qingdao Jindian, China). Each well was seeded with 1 × 10^4^ MC3T3-E1 in 2 mL complete α-MEM medium with 10% (v/v) FBS and 1% penicillin-streptomycin-amphotericin B solution (100×), supplemented with SrHP at a final concentration of 0.5 mg/mL. The cell-laden SrHP complexes were incubated in a humidified 5% CO_2_ atmosphere at 37°C. Medium replacement was performed on Day 3. Cellular adhesion efficiency was evaluated at predetermined intervals (Days 1, 3 and 5) through microscopic observation and quantitative assays.

For HUVECs, substituting α-MEM with complete HG-DMEM medium with 20% (v/v) FBS and 1% penicillin–streptomycin–amphotericin B solution (100×).

### Cell adhesion and spreading morphology

Cellular viability and microcarrier adhesion of M-SrHP and H-SrHP constructs were assessed at 1, 3 and 5 days post-seeding using a Calcein-AM/PI Double Stain Kit solution. Following incubation, cell-laden microspheres were aspirated from ultra-low attachment plates and transferred to standard 48-well plates. After removing the residual complete α-MEM medium, samples underwent two PBS rinses followed by 30 min incubation with 200 μL Calcein-AM/PI working solution under light-protected conditions at 37°C. Fluorescence microscopy (Olympus IX51, Japan) was employed to visualize live (green) and dead (red) cells.

For the evaluation of cell adhesion, M-SrHP and H-SrHP co-cultures were fixed in 4% PFA and permeabilized with 0.1% Triton X-100 on Day 3. The cytoskeletal architecture was labeled with TRITC phalloidin, while nuclei were counterstained with DAPI. Followed by morphological observation using fluorescence microscopy (Olympus IX51, Japan).

### Injectability of cell-laden SrHP

To investigate potential cellular damage during scaffold delivery, M-SrHP and H-SrHP co-culture constructs underwent biomechanical shear stress simulation mimicking clinical injection. Cell-laden SrHP was aspirated from ultra-low attachment plates using a 22-gauge sterile syringe needle (Jiangxi Fenglin, China) and forcibly injected into 48-well tissue culture plates under laminar flow conditions (0.5 mL/s flow rate). Static controls maintained identical culture parameters without mechanical perturbation. Cellular viability was quantitatively assessed and observed by Calcein-AM/PI live-dead fluorescence assays.

After being subjected to a simulated clinical injection, M-SrHP and H-SrHP were seeded into 24-well plates and cell migration was observed 24 h later.

### Angiogenic activity of cell-laden SrHP *in vitro*

The pro-angiogenic effects of M-SrHP, H-SrHP and MH-SrHP were investigated using a wound-healing assay. HUVECs were seeded in 6-well plates at 3 × 10^5^ cells/well and cultured to 90% confluence in a complete HG-DMEM medium. Three uniform linear scratches (width: 500 ± 50 μm) per well were created using a 200 μL sterile pipette tip (Biosharp, China). After removing cellular debris with three gentle PBS washes (pH 7.4), 2 mL of conditioned media (CM-M, CM-H or CM-M&H) were added, with complete HG-DMEM containing 2% FBS serving as the negative control.

After incubating for 12 and 24 h, the wounds were observed and recorded by optical microscope and the quantitative data of the images were processed by ImageJ software.

Pre-cooled Matrigel (CORNING, USA) was coated into 24-well plates, followed by cell seeding at a density of 150 000 cells per well. After 6 h of culture at 37°C with 5% CO_2_, the samples were stained and observed. Images were then quantitatively analyzed using ImageJ software to determine the relative segment length, relative number of meshes and relative number of junction points of the tubes.

### Osteogenic differentiation ability of cell-laden SrHP *in vitro*

Conditioned media (CM) were harvested from three experimental configurations: M-SrHP-derived CM (CM-M) and H-SrHP-derived CM (CM-H) were generated after being cultured in complete medium (α-MEM for MC3T3-E1, HG-DMEM for HUVECs) for 5 days and then subjected to 24-h serum deprivation in basal media. The culture medium was collected by filtration through a 0.22 μm membrane. To obtain a co-culture-conditioned medium (CM-MH), M-SrHP and H-SrHP were co-seeded at a 1:1 ratio for 3 days, then maintained in a 1:1 (v/v) hybrid medium of complete α-MEM and HG-DMEM medium for 48 h, then, subjected to identical serum deprivation and filtration protocols.

To evaluate the osteogenic potential of M-SrHP, H-SrHP and MH-SrHP, MC3T3-E1 were seeded into 24-well plates at a density of 1 × 10^4^ cells per well. After 3 days, cells were exposed to osteogenic induction medium (OIM) containing 0.1 μM dexamethasone, 50 μg/mL L-ascorbic acid and 10 mM β-glycerophosphate, supplemented with 20% (v/v) conditioned media (CM-M, CM-H or CM-M&H). Alkaline phosphatase (ALP) activity was assessed on Day 7 using BCIP/NBT staining, while extracellular matrix mineralization was quantified on Day 14 *via* Alizarin Red S (ARS) staining. Stained specimens were imaged under bright-field microscopy (Olympus IX51). Three random fields were selected, and triplicate measurements from these fields were analyzed using ImageJ software (v1.53q, NIH, USA) to quantify the stained area.

### Inhibiting osteoclast differentiation ability of cell-laden SrHP *in vitro*

The anti-osteoclastogenic potential of cell-laden SrHP was evaluated by TRAP staining. RAW264.7 were seeded at 8 × 10³ cells per well in 24-well plates and cultured in a complete α-MEM medium. The complete α-MEM medium was replaced with osteoclastogenic induction medium (50 ng/mL RANKL) supplemented with 20% (v/v) conditioned media (CM-M, CM-H or CM-M&H), while controls received induction medium without CM supplementation. After 5 days of differentiation, cells were fixed in 4% paraformaldehyde and incubated with TRAP staining solution for 2 h at 37°C protected from light. Following three washes with PBS, TRAP-positive cells were analyzed using bright-field microscopy. The stained area was quantified using ImageJ software.

### Gene expression

The osteo-inductive, angiogenic and anti-osteoclastic capacities of M-SrHP, H-SrHP and MH-SrHP were systematically assessed through quantitative reverse transcription polymerase chain reaction (qRT-PCR).

MC3T3-E1 (3 × 10^5^ cells/well) were cultured in HG-DMEM supplemented with 10% FBS and conditioned media (CM-M, CM-H or CM-M&H) for 7 days. HUVECs (3 × 10^5^ cells/well) were exposed to identical media formulations for 3 days. RAW264.7 monocytes (3 × 10^5^ cells/well) were differentiated into osteoclasts *via* RANKL (50 ng/mL) induction for 5 days. Total RNA was extracted according to instructions given in the RNA extraction kit (Trizol, Thermo Fisher, USA) and the PCR reaction was carried out on a CFX96 PCR system (Bio-Rad, USA) according to the instructions given in the SYBR Premix Ex Taq™ kit (Biosharp, China). Glyceraldehyde-3-phosphate dehydrogenase (*GAPDH*) served as the endogenous control. Relative expression was calculated *via* the 2^−ΔΔCt^ method. The primer sequences of osteogenesis-related genes (*BMP-2, ALP, COL-1, OCN)*, angiogenesis-related genes (*VEGF, ANG-1)* and osteoclast-related genes (*TRAP, MMP-9, CTSK)* were shown (Supplementary Tables S1–S3).

### Statistical analysis

All data represent the mean ± standard deviation (SD) from three independent experiments. SPSS software (Version 19, IBM, USA) was used for statistical analysis. Data were analyzed by one-way ANOVA with Tukey’s test (*P < 0.05, **P < 0.01, ***P < 0.001 and ****P < 0.0001 were defined as statistically significant).

## Results and discussions

### Material characteristics of SrHP

**Figure 2:**
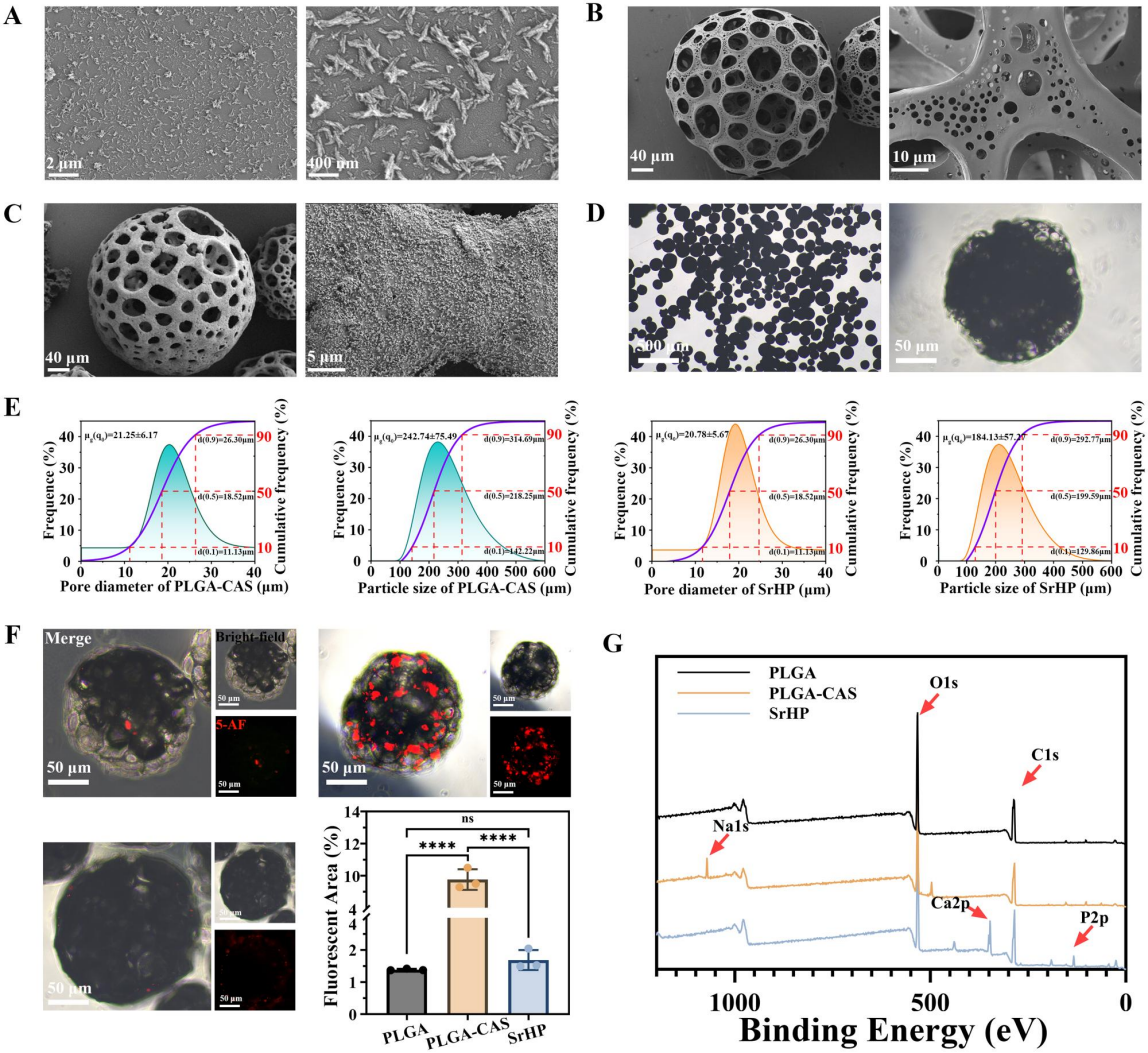
Characteristics of cage-like structures. (**A**) SEM images of needle-like Sr-HA; (**B**) SEM images of porous spherical PLGA-CAS; (**C**) SEM images of SrHP shows PLGA-CAS structures successfully loaded with needle-like Sr-HA. (**D**) Optical microscopy images of SrHP exhibiting a regular spherical shape. (**E**) Pore diameter and particle size distribution of PLGA-CAS and SrHP: frequency-based nanoparticles size distribution and cumulative distribution with the geometric mean diameter *μ*_g_(*q*_0_) and the characteristic diameters d0.1, d0.5 and d0.9. (**F**) 5-Aminofluorescein staining images and quantitative analysis of PLGA, PLGA-CAS and SrHP (*n* = 3, data represent mean ± SD). (**G**) XPS survey spectra of PLGA, PLGA-CAS and SrHP. ****P < 0.0001; ns, not significant.

Sr-HA has good dispersibility, presenting a needle-like shape and its size is mainly distributed around 200 nm (Figure 2A). The PLGA-CAS exhibited a porous spherical architecture with interconnected internal channels, as evidenced by SEM imaging (Figure 2B). SEM images of SrHP demonstrate the successful deposition of HA onto alkali-treated PLGA surfaces (Figure 2C). Under optical microscopy, SrHP displayed regular spherical shapes (Figure 2D). The magnified view shows a uniform porous structure throughout the microspheres, which, together with the SEM images, demonstrates the successful synthesis of cage-like microspheres.

The geometric mean pore sizes were measured as 21.25 ± 6.17 μm for PLGA-CAS and 20.78 ± 5.67 μm for SrHP, demonstrating that both materials exhibited a pore size greater than 20 μm. Such a pore dimension meets the critical threshold for facilitating cellular infiltration and nutrient exchange, thereby fulfilling the essential criteria for their application as cell-delivery vehicles [53]. In particle size analysis, the geometric mean diameters were 242.74 ± 75.49 μm for PLGA-CAS and 184.13 ± 57.27 μm for SrHP, respectively, indicating a reduction in particle size for the SrHP composite (Figure 2E). This size decrease in SrHP aligns with reported ethanol-induced PLGA contraction during Sr-HA loading [54].

Post-alkali treatment induced ester group hydrolysis on PLGA-CAS surfaces, generating carboxyl groups that facilitated Sr-HA immobilization through Ca^2+^ chelation [55, 56]. Fluorescence quantification demonstrated a trend of low-high-low intensity among PLGA, alkali-treated PLGA-CAS and Sr-HA-loaded SrHP, respectively (Figure 2F). This pattern, visualized via red fluorescence (*λ*_ex_ = 495 nm) from 5-aminofluorescein bound specifically to surface carboxyl groups, confirms that alkali treatment exposed carboxyl groups by hydrolyzing ester bonds and subsequent Sr-HA loading reduced their availability through Ca^2+^ chelation [57]. NaOH treatment hydrolyzed ester bonds, followed by ion exchange during HA loading, as proven by XPS survey spectra. The spectra showed a transient Na signal in PLGA-CAS (from formed sodium carboxylates), which disappeared in SrHP concurrent with the appearance of Ca/P signals, evidencing Na^+^ displacement by Ca^2+^ via chelation [58]. This verifies the generation of carboxyl groups after NaOH treatment and demonstrates the strong binding between HA and PLGA (Figure 2G).

**Figure 3:**
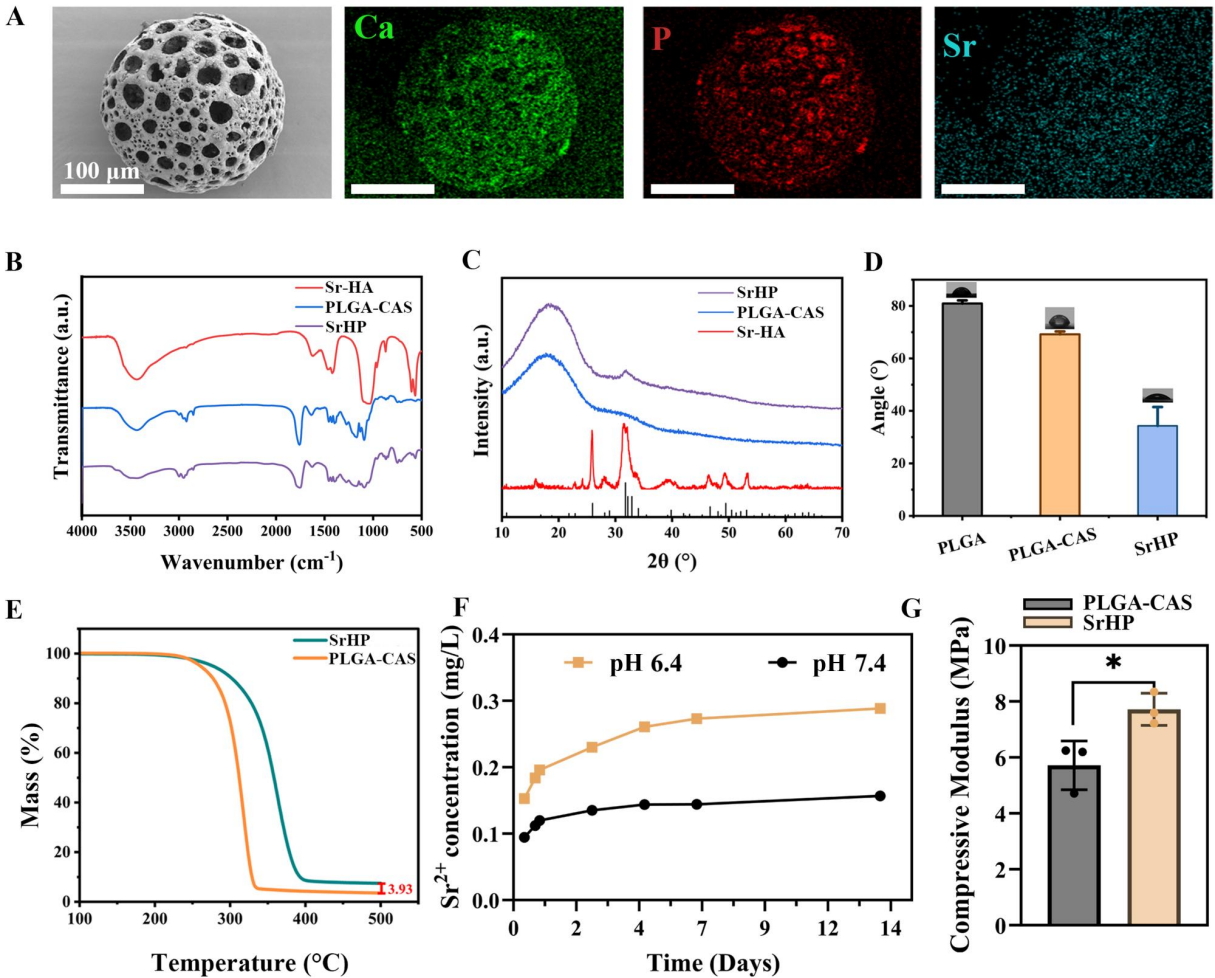
Physical characterization of cage-like structures. (**A**) EDS elemental mapping of SrHP showing the uniform spatial distribution of P, Ca, and Sr on the microsphere surface; (**B**) FTIR spectra of SrHP, PLGA-CAS and Sr-HA; (**C**) XRD patterns of SrHP, PLGA-CAS and Sr-HA; (**D**) Images of water contact angles demonstrating the sequentially enhanced hydrophilicity of PLGA-CAS, AT-PLGA-CAS, and SrHP (*n* = 3, data represent mean ± SD); (**E**) Thermal decomposition curves presenting the temperature-dependent mass alterations of PLGA-CAS and SrHP; (**F**) Release curve of Sr^2+^ from SrHP in PBS at pH 7.4 and 6.4 over 14 days.(**G**) Compressive modulus of PLGA-CAS and SrHP (*n* = 3, data represent mean ± SD). *P < 0.05.

The elemental distribution and composition of SrHP were analyzed using EDS. A uniform spatial dispersion of phosphorus (P), calcium (Ca) and Sr across the microsphere surface confirmed the homogeneous loading of Sr-HA on PLGA-CAS (Figure 3A and Supplementary Figure S1).

In the FTIR spectrum of Sr-HA, the PO_4_³^−^ ν_2_ bending modes appear distinctly at 564 and 602 cm^−1^, consistent with O–P–O bending vibrations in phosphate minerals, while the ν_3_ stretching modes are observed at 985, 1034 and 1108 cm^−1^. The SrHP spectrum displays these same absorptions at identical wavenumbers, confirming the preservation of phosphate groups within the SrHP matrix. PLGA-CAS exhibits a pronounced ester carbonyl (C = O) stretching band at 1759 cm^−1^, which is likewise present in the SrHP spectrum, indicating that carbonyl functionalities remain intact following composite formation (Figure 3B). The perfect alignment of Sr-HA and PLGA-CAS characteristic peaks within the SrHP spectrum, thus, demonstrates the successful integration of both inorganic phosphate and polymeric carbonyl moieties into the SrHP framework.

XRD patterns of Sr-HA matched those of standard hydroxyapatite (HA; JCPDS No. 09-0432), exhibiting sharp reflections at 2θ = 25.9° (002), 31.8° (211) and 32.1° (112), confirming that the HA lattice remained intact following Sr^2+^ incorporation. PLGA-CAS displayed a broad, amorphous halo between 10° and 25° 2θ, characteristic of its amorphous polymeric nature. In the SrHP composite, the broad amorphous halo from PLGA-CAS coexisted with distinct hydroxyapatite (HA) reflections at *2*θ ≈ 31°–35°, confirming the simultaneous presence of non-crystalline polymer and crystalline calcium phosphate phases (Figure 3C). These dual-phase XRD features demonstrate that SrHP successfully integrates amorphous PLGA-CAS and crystalline Sr-HA without compromising the structural integrity of either component [59].

To further verify successful hydroxyapatite synthesis, the crystal structure of Sr-HA constituting its primary phase was analyzed by transmission electron microscopy (TEM). High-resolution TEM (HRTEM) images and corresponding diffraction rings revealed well-defined lattice fringes with an interplanar spacing of 3.483 Å. This value is slightly larger than the standard (002) crystallographic plane (3.44 Å) of pure hydroxyapatite (JCPDS No. 09-0432), attributed to the lattice expansion caused by the substitution of larger Sr^2+^ for Ca^2+^ (Supplementary Figure S2). Furthermore, indexing of the diffraction rings confirmed their assignment to characteristic HA lattice planes, verifying successful synthesis of Sr-HA nanoparticles. Both XRD patterns and TEM-analyzed crystal structures collectively confirm successful synthesis of hydroxyapatite, providing conclusive evidence of phase formation.

Surface hydrophilicity critically influences cell-material interactions, particularly adhesion when materials serve as cell carriers. In this study, we measured static water contact angles of PLGA-CAS, alkali-treated PLGA-CAS (AT-PLGA-CAS) and the SrHP using a contact-angle goniometer. PLGA-CAS exhibited a contact angle of 81°, which decreased modestly following alkaline treatment of the PLGA backbone. Remarkably, the SrHP composite incorporating Sr-HA showed a drastically reduced contact angle of 34° (Figure 3D), reflecting its markedly enhanced hydrophilicity. This pronounced hydrophilicity arises from abundant surface hydroxyl and phosphate moieties contributed by Sr-HA, which are known to promote water affinity. These findings are consistent with those reported by Yu *et al.* [5], confirm that the SrHP scaffold attains the hydrophilic profile requisite for robust cell adhesion when deployed as a carrier.

TGA showed that neat PLGA fully decomposes by 500°C, whereas Sr-HA remains thermally stable under the same conditions. By comparing the residual mass of SrHP with that of PLGA-CAS after complete polymer decomposition, the Sr-HA content in SrHP was determined to be 3.93 wt%. Both materials follow similar mass‐loss trajectories and reach a plateau between 300 and 350°C; However, the decomposition onset of SrHP occurs at a higher temperature than that of PLGA-CAS, indicating that the incorporation of Sr-HA delays polymer degradation and enhances thermal stability (Figure 3E).

Previous studies have demonstrated that low-level Sr^2+^ release enhances osteoblast differentiation and inhibits osteoclast activity, whereas excessive Sr^2+^ can impair bone mineralization and compromise osteoporosis therapy [60]. To simulate the local acidic microenvironment of osteoporotic bone defects, we evaluated the Sr^2+^ release kinetics of SrHP in PBS at both physiological (pH 7.4) and acidic (pH 6.4) levels. ICP-MS analysis over 14 days revealed a biphasic release profile in both media: an initial burst release within the first 24 h, followed by a transition to stable, sustained release after 3 days (Figure 3F). This sustained pattern ensures prolonged ion availability within therapeutic windows to foster osteogenesis. Notably, the cumulative release of Sr^2+^ was significantly accelerated in the acidic medium (pH 6.4). This pH-responsive behavior suggests an intelligent ‘on-demand’ delivery mechanism, where elevated Sr^2+^ concentrations are liberated specifically in response to the acidic resorptive environment to effectively inhibit osteoclast activity, while the basal release supports continuous bone formation.

The compressive modulus of PLGA-CAS and SrHP was derived from the stress–strain curves (Figure 3G and Supplementary Figure S3). The analysis indicated that both materials possessed adequate mechanical stability. Among them, SrHP, with its smaller microsphere size, exhibited greater compressive strength, suggesting that the incorporation of Sr-HA did not compromise the mechanical properties of PLGA. The injectable bone repair unit features a porous structure to facilitate cell infiltration while also providing adequate compressive properties to stabilize the defect site and deliver mechanical stimulation that promotes regeneration [61, 62].

### Biocompatibility of SrHP

The hemolytic properties of SrHP were assessed using rat tail vein blood, revealing hemolysis rates below 1% at concentrations of 0.25, 0.5 and 1 mg/mL, indicating excellent hemocompatibility (Supplementary Figure S4).

**Figure 4:**
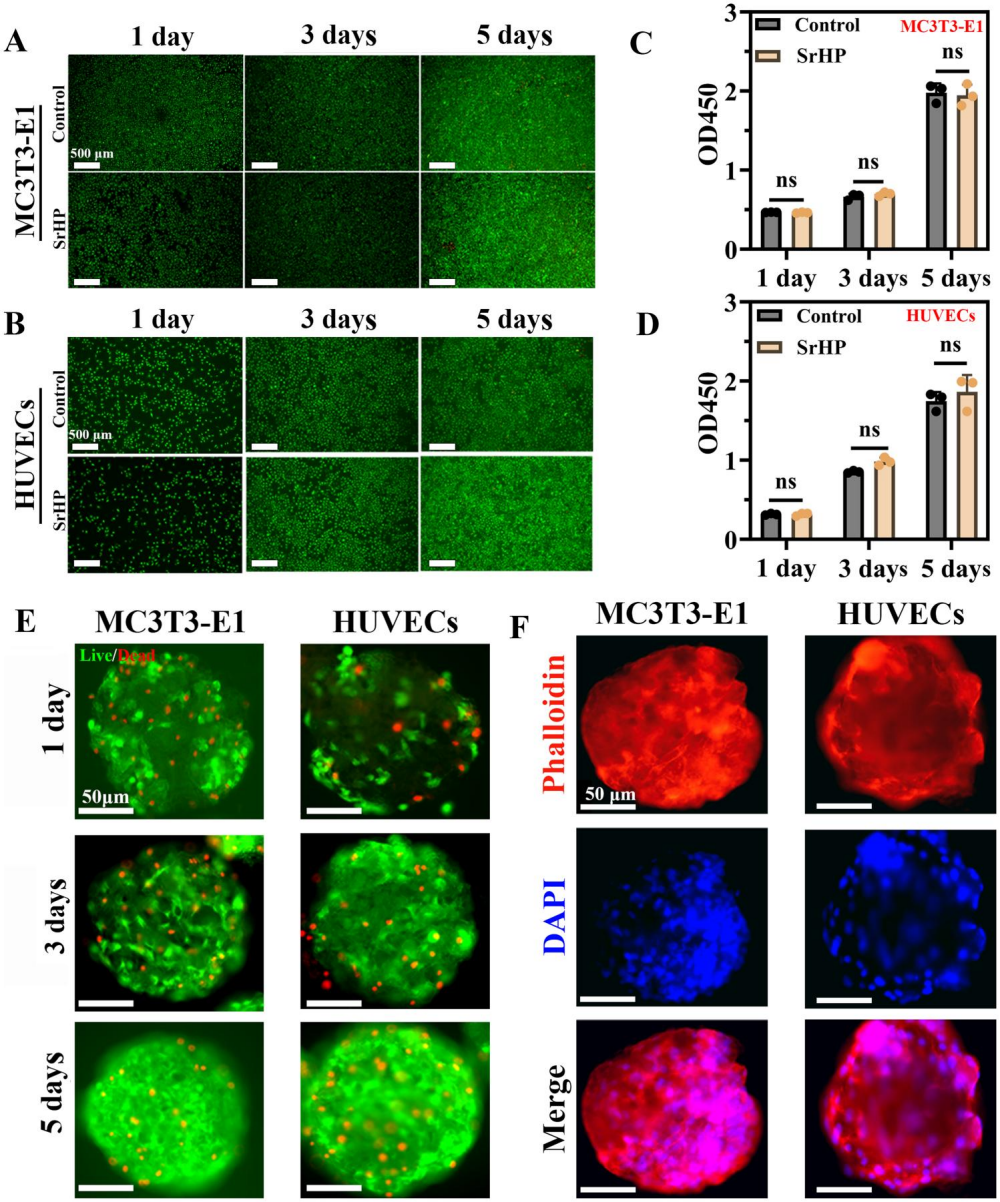
Cytotoxicity and cell proliferation of SrHP. Live/dead fluorescence images confirming the absence of cytotoxicity in (**A**) MC3T3-E1 and (**B**) HUVECs co-cultured with SrHP after 1, 3 and 5 days; Proliferation quantification of (**C**) MC3T3-E1 and (**D**) HUVECs co-cultured with SrHP after 1, 3 and 5 days, with no significant difference observed (*n* = 3, data represent mean ± SD); (**E**) Live/dead fluorescence staining images of MC3T3-E1 and HUVECs adhered to the surface of SrHP, demonstrating uniform coverage and robust viability over 5 days; (**F**) Nuclear and cytoskeletal staining of MC3T3-E1 and HUVECs adhered to the surface of SrHP reveals extensive cell spreading, with cytoskeletal filaments conforming tightly to the scaffold surface. ns, not significant.

SrHP exhibited excellent cytocompatibility: live/dead fluorescent staining confirmed high cell survival rates in SrHP-treated cultures (Figure 4A and B), which was further supported by CCK-8 assays showing cell viabilities above 80% throughout the culture period. No significant viability difference was observed between SrHP co-culture and cell-only controls (*P* > 0.05, NS). Additionally, proliferation kinetics were indistinguishable between control and SrHP-treated cells at every time point, confirming the absence of cytotoxic effects (Figure 4C and D).

The surface morphology and cytocompatibility of SrHP were evaluated by seeding MC3T3-E1 and HUVECs onto the scaffold, followed by assessments of cell viability, adhesion and pseudopodial extension. Live/dead assays of cells seeded on microspheres for 3 days demonstrated uniform coverage and robust viability. Consequently, Day 3 was selected as the time point for subsequent analyses (Figure 4E). Fluorescence micrographs of DAPI-stained nuclei (blue) and TRITC-phalloidin-labeled F-actin (red) showed that by Day 3, both MC3T3-E1 and HUVECs had spread extensively, with cytoskeletal filaments conforming tightly to the scaffold surface and nuclei distributed evenly across the SrHP (Figure 4F). These findings confirm that SrHP supports firm cell adhesion and relevant cytoskeletal organization, underscoring its suitability as a cell-carrier scaffold.

### Cell morphology and injectability of cell-laden SrHP

**Figure 5:**
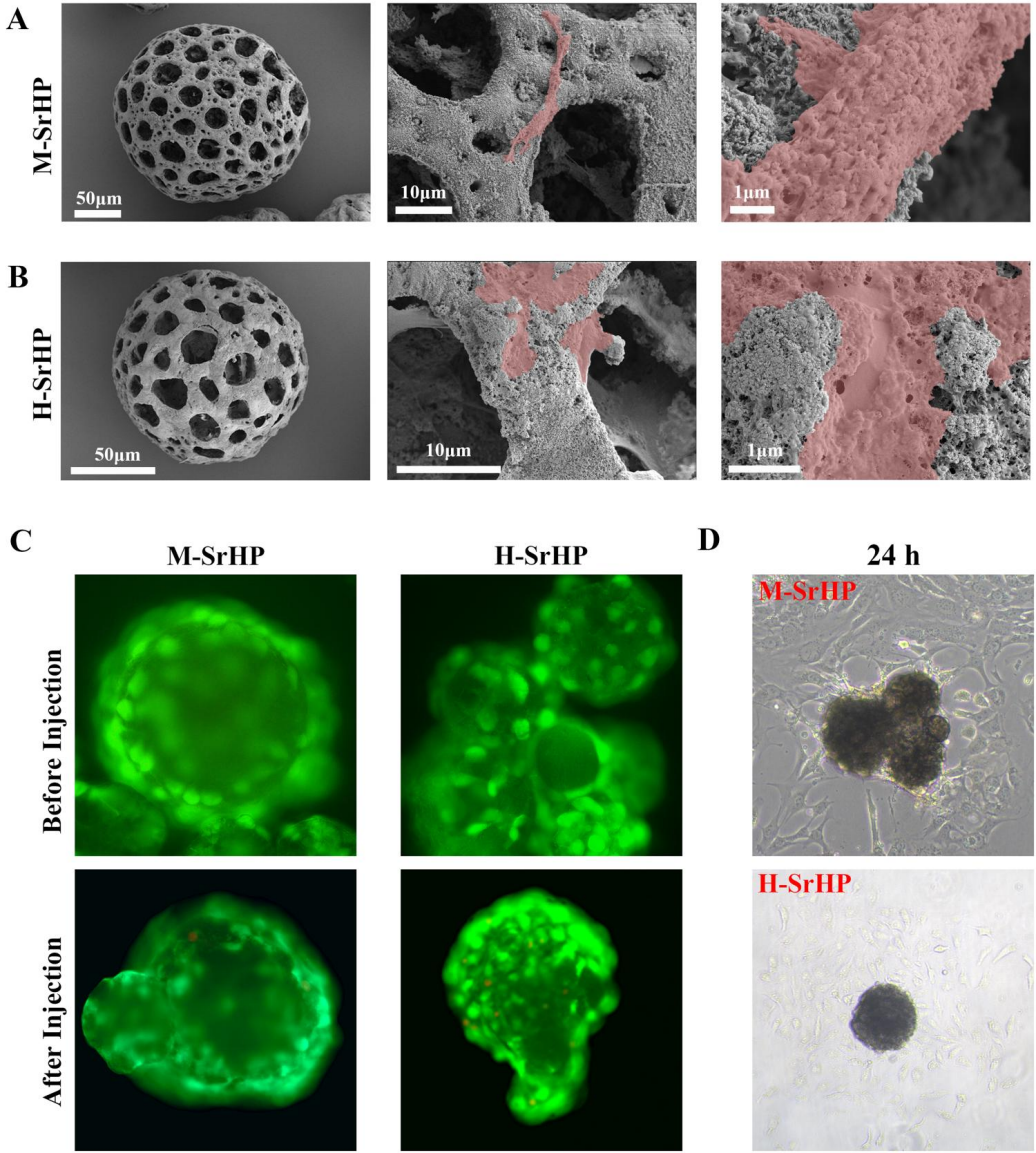
The cytoprotective effect of SrHP. SEM images of (**A**) M-SrHP and (**B**) H-SrHP, with magnified views on the right demonstrating that the cells maintain their characteristic morphology without compromising the porous architecture of the microspheres. (**C**) Live/dead staining of M-SrHP and H-SrHP before and after injection, demonstrating that the cells maintain high viability post-injection; (**D**) The outgrowth of MC3T3-E1 cells and HUVECs was monitored at 24 h after seeding with M-SrHP and H-SrHP, confirming the retention of normal cellular functions.

SEM analysis revealed that cell adhesion did not compromise the porous architecture of the microspheres, with well-defined cellular features of MC3T3-E1 and HUVECs visible on the surface (Figure 5A and B). MC3T3-E1 maintained characteristic spindle-shaped morphology, consistent with standard *in vitro* observations, indicating that the microstructured-to-nanostructured Sr-HA/PLGA surface supports normal cellular physiological functions [63].

As an injectable cell‐delivery vehicle, SrHP preserved both its structural integrity during injection and the viability of surface‐adherent cells (Figure 5C). Live/dead fluorescence staining showed negligible differences in nonviable cell density between injected and noninjected groups. SrHP can provide sustained high cell viability upon delivery. These findings are consistent with the protective effects observed in shape-memory injectable hydrogel carriers [64].

At 24 h after seeding M-SrHP and H-SrHP, MC3T3-E1 cells and HUVECs exhibited outward migration. Notably, individual cells were observed straddling the interface, with one portion remaining within the microsphere and another portion extended onto the culture plate. All migrated cells maintained normal proliferation (Figure 5D). These findings confirm the effectiveness of this osteogenic microsphere system in protecting encapsulated cells and sustaining their function.

### Angiogenic activity of cell-laden SrHP *in vitro*

**Figure 6:**
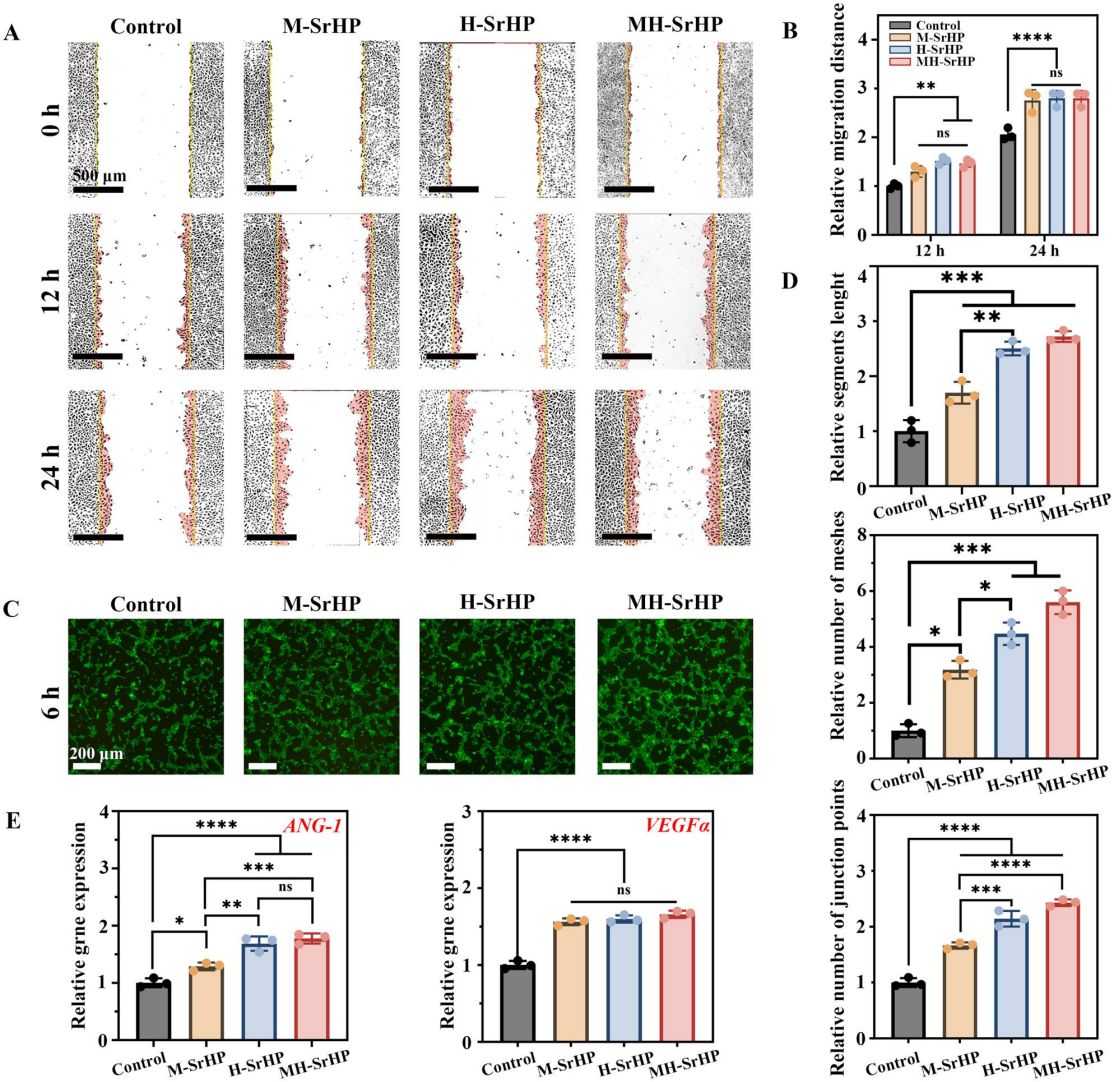
Angiogenic activity of cell-laden SrHP *in vitro*. Representative images (**A**) and quantitative analysis (**B**) of HUVEC migration in a scratch wound-healing assay after 0, 12, and 24 h of culture with extracts from M-SrHP, H-SrHP, and MH-SrHP, demonstrating enhanced endothelial cell motility, particularly in the H-SrHP and MH-SrHP groups (*n *= 3, data represent mean ± SD); Tube formation assay (**C**) of HUVECs with the respective conditioned media for 6 h, and the corresponding quantification (**D**) of relative segment length, number of meshes, and junction points, demonstrating that cell loading, particularly with HUVECs, led to the formation of significantly denser mesh-like vascular networks (*n *= 3, data represent mean ± SD); (**E**) relevant mRNA (*ANG-1, VEGFα*) expression of HUVECs co-cultured with cell-laden SrHP for 3 days was analyzed by qRT-PCR (*n *= 3, data represent mean ± SD). *P < 0.05, **P < 0.01, ***P < 0.001 and ****P < 0.0001. ns, not significant.

As bone defects frequently disrupt local vascular networks and impair nutrient delivery and waste removal, promoting angiogenesis and accelerating vascular reconstruction are essential for effective bone regeneration [65–67]. Endothelial cell migration represents a pivotal step in neovessel formation [68–70]. HUVEC migration across three microsphere systems (M-SrHP, H-SrHP and MH-SrHP) was evaluated via wound-healing assays, with migration distances quantified using ImageJ at 12 and 24 h time points. Both H-SrHP and MH-SrHP significantly enhanced HUVECs’ motility at 12 h, while all cell-laden SrHP groups outperformed controls by 24 h, demonstrating their pro‐angiogenic potential (Figure 6A and B). HUVECs were seeded on Matrigel and treated with three types of conditioned media (M-SrHP, H-SrHP and MH-SrHP) for 6 h to assess tube formation. Compared to the control group, all three treatment groups developed a denser mesh-like network (Figure 6C). Quantitative analysis of the tube formation assay revealed that the H-SrHP and MH-SrHP groups exhibited the most significant pro-angiogenic effects, as indicated by the relative segment length, relative number of meshes and relative number of junction points (Figure 6D). These findings are consistent with the results from the scratch assay.

Analysis of HUVECs revealed significant upregulation of angiogenic markers *ANG-1* and *VEGFα* when cultured with cell-laden SrHP microspheres compared to untreated controls, as demonstrated by qRT-PCR. Notably, *VEGFα* and *ANG-1* transcript levels in H-SrHP and combined MH-SrHP groups exceeded those in M-SrHP, indicating that endothelial cells on SrHP activate the *VEGFα*/*ANG-1* signaling pathway (Figure 6E). These transcriptional changes are similar to the accelerated HUVEC migration observed in scratch assays, confirming the growth factors released by endothelial cells loaded in SrHP exhibit favorable effects on the formation and stabilization of new blood vessels [71].

Prasadam *et al.* [72] pointed out that the osteocyte lacunar-canalicular network is closely associated with blood vessels, with osteocyte dendrites directly connecting to the vascular walls within the bone matrix. Meanwhile, studies have shown that osteocytes can regulate angiogenesis through the release of paracrine signals [73]. This suggests that osteocytes may interact closely with bone endothelial cells. In this study, similar conclusions can be drawn: M‑SrHP alone exhibits limited pro‑angiogenic effects, whereas its combination with H‑SrHP significantly enhances vascular repair in bone defects.

### Osteogenic differentiation ability of cell-laden SrHP *in vitro*

**Figure 7:**
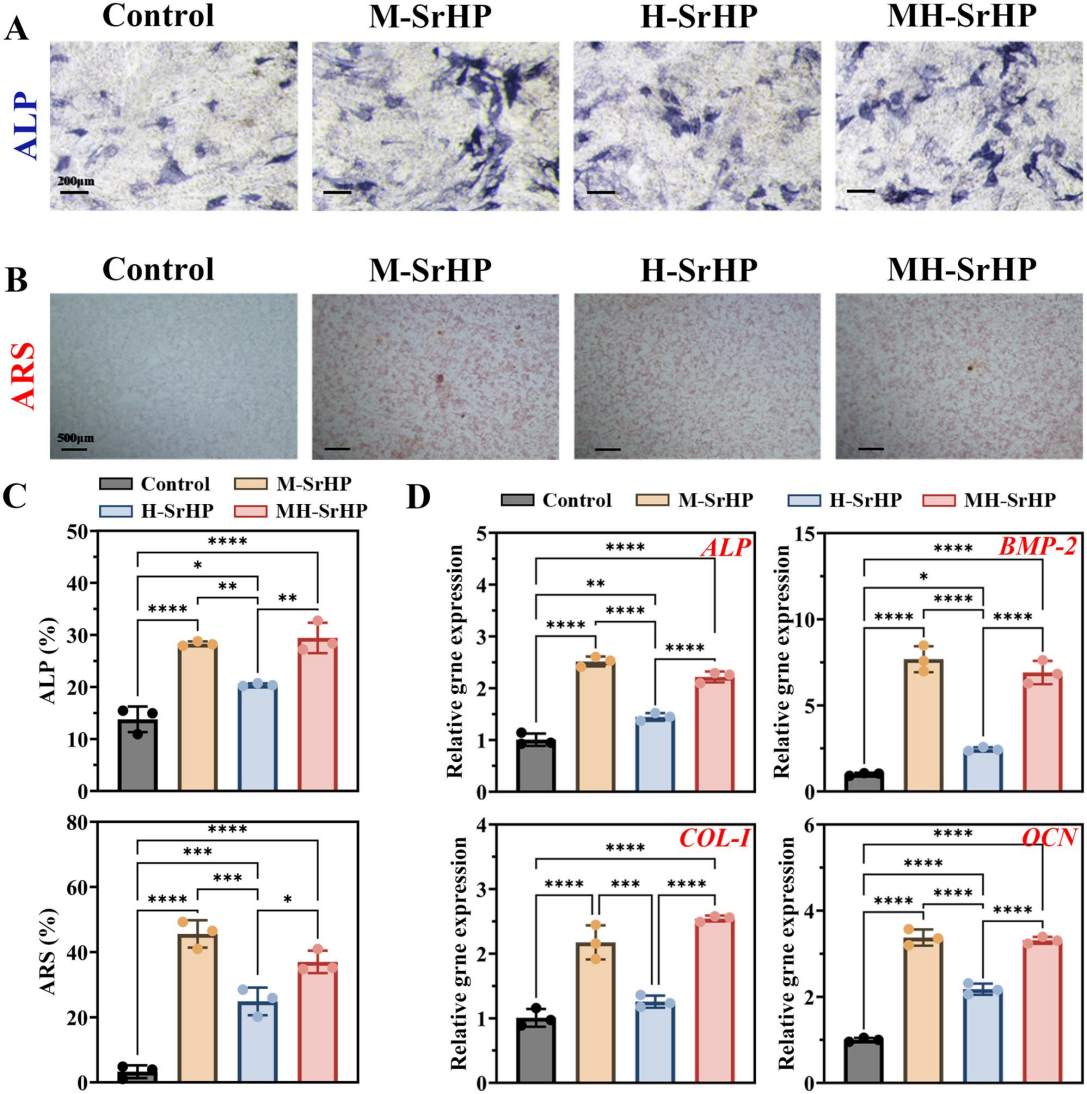
Osteogenic differentiation ability of cell-laden SrHP *in vitro*. (**A**) ALP staining of MC3T3-E1 after 7 days of culturing, demonstrating that cell loading, particularly with MC3T3-E1, significantly promoted ALP expression; (**B**) ARS staining of MC3T3-E1 after 14 days of culturing, demonstrating that cell loading, particularly with MC3T3-E1, promoted calcium nodule deposition; (**C**) Quantitative analysis of the stained area in ALP and ARS staining, demonstrating that cell loading, particularly with MC3T3-E1, significantly promoted osteogenic differentiation (*n *= 3, data represent mean ± SD). (**D**) Relevant mRNA (*ALP, BMP-2, Col-1, OCN*) expression of MC3T3-E1 co-cultured with cell-laden SrHP for 7 days was analyzed by qRT-PCR (*n *= 3, data represent mean ± SD). *P < 0.05, **P < 0.01, ***P < 0.001 and ****P < 0.0001. ns, not significant.

ALP activity and calcium nodule formation, which are hallmarks of osteogenic differentiation, were assessed by ALP staining and ARS assays. Both assays showed minimal staining in the control group, whereas the M-SrHP, H-SrHP and MH-SrHP groups exhibited markedly enhanced osteogenic responses (Figure 7A and B). Quantitative analysis indicated that M-SrHP exhibited the strongest ALP signal and the largest mineralized nodules, reflecting its high concentration of bioactive factors derived from osteoblasts. Notably, H-SrHP also enhanced osteogenic markers relative to control, confirming that endothelial cell-secreted factors support (Figure 7C) [74].

To further evaluate the osteogenic potential of cell-laden SrHP constructs, the mRNA expression levels of *OCN*, *ALP*, *BMP-2* and *COL-I* in MC3T3-E1 were quantified by qRT-PCR. All cell-laden SrHP groups showed significant upregulation of these osteogenic markers compared to the unmodified control (*P* < 0.05). Notably, the M-SrHP and MH-SrHP groups exhibited markedly higher transcriptional levels than the H-SrHP group, indicating that signals derived from osteoblasts amplify the osteoinductive effect mediated by Sr-HA (Figure 7D).

These findings suggest that the inclusion of MC3T3-E1 enhances the local release of bioactive factors, promoting autocrine/paracrine mechanisms that substantially boost osteogenic differentiation within the Sr-HA/PLGA scaffold [75].

### Inhibiting osteoclast differentiation ability of cell-laden SrHP *in vitro*

**Figure 8:**
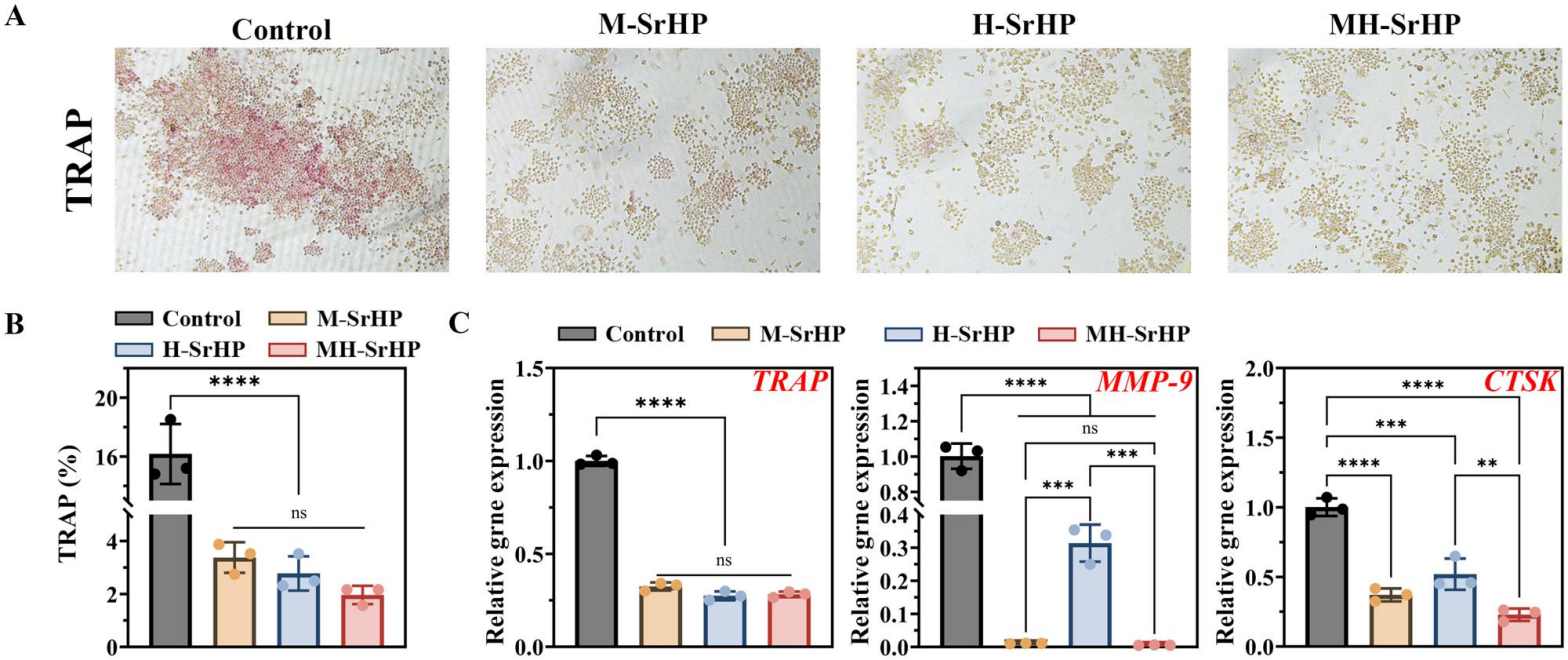
Inhibiting osteoclast differentiation ability of cell-laden SrHP *in vitro*. Representative images of TRAP staining (**A**) and the corresponding quantitative analysis (**B**) of RAW 264.7 macrophages cultured with extracts from M-SrHP, H-SrHP, and MH-SrHP for 5 days in the presence of RANKL demonstrate a virtual absence of TRAP-positive cells in all treatment groups, indicating effective inhibition of osteoclastogenesis compared to the control (*n *= 3, data represent mean ± SD). (**C**) Relevant mRNA (*TRAP, MMP-9 and CTSK*) expression of RAW 264.7 cells co-cultured with cell-laden SrHP for 5 days were analyzed by qRT-PCR (*n *= 3, data represent mean ± SD). *P < 0.05, **P < 0.01, ***P < 0.001 and ****P < 0.0001. ns, not significant.

TRAP staining revealed a prominent population of TRAP-positive osteoclasts in the control group, whereas the M-SrHP, H-SrHP and combined MH-SrHP treatments showed virtually no TRAP-positive cells (Figure 8A and B). TRAP activity is widely recognized as a specific cytochemical marker for osteoclast identification and bone resorption assessment, and its near-absence in all cell-laden SrHP groups indicates effective inhibition of osteoclastogenesis. By suppressing osteoclast formation, these materials hold significant promise for attenuating pathological bone resorption in osteoporosis.

To further elucidate the anti-osteoclastic potential of cell-laden SrHP, RAW 264.7 macrophages were cultured for 5 days in extracts from M-SrHP, H-SrHP and combined MH-SrHP in the presence of RANKL, and gene expression of *TRAP*, *MMP-9* and *CTSK* was quantified by qRT-PCR. All cell-laden SrHP extracts markedly suppressed transcript levels of these osteoclast-specific markers compared to controls.

Although Sr^2+^ ions provided fundamental osteoclast suppression in all groups, M-SrHP and MH-SrHP elicited significantly stronger inhibition. This cell-dependent enhancement suggests that synergistic interactions between Sr^2+^ and factors secreted by MC3T3-E1 enhance the inhibition of osteoclast differentiation and point toward a novel therapeutic strategy (Figure 8C) [76].

## Discussion

The customizable, multifunctional cell-laden bone repair units hold great promise in the field of regenerative medicine. The MH‑SrHP system designed in this study demonstrates significant potential for repairing osteoporotic bone defects. The combination of Sr^2+^ with HA not only enables the sustained release of Sr^2+^ but also endows HA with dual functions of osteoinduction and osteoclast inhibition. By utilizing this composite to modify the surface of PLGA‑CAS, we not only altered its hydrophilicity but also introduced biofunctionalization to the material. The strategic integration of Sr^2+^, HA and PLGA forms a cell carrier with excellent biocompatibility and cellular regulatory capabilities. Unlike conventional materials focused solely on bone repair, this study simultaneously loaded both MC3T3-E1 cells and HUVECs. Loading MC3T3-E1 cells accelerates the bone repair process, while loading HUVECs endows the material with pro‑angiogenic ability, thereby achieving multifunctional effects that promote osteogenesis, inhibit osteoclast activity and stimulate angiogenesis. Furthermore, the synergy between the material and the cells, as well as the crosstalk between osteoblasts and endothelial cells, leads to a significant enhancement of overall functionality. Therefore, the dual-cell bone repair unit developed in this study holds promise for addressing current challenges in bone regeneration within osteoporotic defects, offering a prospective solution to the difficulties encountered in the field of aged bone repair.

Compared to commonly used ion-doped bone repair scaffolds, such as Zn^2+^- or Sr^2+^-modified titanium surfaces [37, 77]. The 3D porous structure of the microspheres not only protects the encapsulated cells upon implantation into the defect site but also facilitates intercellular material exchange and communication. Notably, crosstalk between different cell types plays a critical role in the bone repair process [78]. The generation of new blood vessels supplies oxygen to cells in the defect area, accelerating the bone repair process. In turn, osteoblasts secrete pro‑angiogenic factors that stimulate endothelial cells to form new vascular structures [79]. Therefore, this reciprocal interplay contributes to accelerated repair of the bone defect site and a shortened treatment period.

Despite showing promising potential for clinical application in osteoporotic bone repair, this study recognizes that significant limitations to its translation remain. First, the long‑term *in vivo* safety of cell carriers has consistently been one of the central concerns in cell therapy. Although the microsphere‑based bone repair unit can effectively reduce the surgical area and minimize secondary injury during implantation, it remains difficult to completely eliminate bacteria latent in hair follicles and sebaceous glands during surgery [80, 81]. Additionally, the static culture environment cannot fully mimic the *in vivo* conditions. The application of biomimetic bioreactors could help address this limitation by providing simulations of both dynamic mechanical loading and static substrate environments [82, 83]. Scalable production of injectable microspheres has long been a major hurdle for clinical translation. While studies have shown that PLGA drug-loaded microspheres can be fabricated in a continuous manner using an in-line homogenizer, the scalable production of porous microspheres remains a significant challenge with limited progress [84]. Addressing the triple challenge of achieving scale-up synthesis, conferring antibacterial and anti-inflammatory properties and upgrading the microsphere culture system will be central to subsequent research.

## Conclusion

PLGA-CAS was fabricated using a W_1_/O/W_2_ double-emulsion technique to create porous microspheres; needle-like Sr-HA, synthesized via wet-chemical precipitation, was then uniformly applied to form the composite SrHP scaffold. Incorporation of the nanostructured Sr-HA significantly increased the hydrophilicity of the PLGA-CAS, as evidenced by reduced water contact angles and improved surface wettability. ICP-MS analysis demonstrated a sustained release profile of Sr^2+^, which has been shown to promote osteoblastic differentiation and inhibit osteoclast differentiation over an extended time frame. Cells were loaded onto SrHP in three configurations: MC3T3-E1 alone, HUVECs alone and a 1:1 co-culture of both cell types. In addition, the viability of the cells seeded on SrHP and the injectability of cell-laden SrHP were evaluated. Functionally, both M-SrHP and the co-culture MH-SrHP significantly enhanced osteogenic markers in MC3T3-E1, while H-SrHP and MH-SrHP promoted endothelial migration and upregulated angiogenic gene expression in HUVECs. Additionally, all cell-laden SrHP constructs effectively suppressed osteoclast differentiation in RAW264.7 cultures. Collectively, these multifunctional microspheres meet the essential requirements for osteogenesis, angiogenesis and inhibition of osteoclast differentiation, thereby offering a promising strategy for repairing osteoporotic bone defects and restoring bone homeostasis within osteoporotic microenvironments.

## Supplementary Material

rbag051_Supplementary_Data

## Data Availability

The data that support the findings of this study are available from the corresponding author upon reasonable request.
