# Supplementary material for: The Sr-HA-loaded PLGA cage structure combines cells to construct a bone tissue repair unit
Source: Regen Biomater. 2026 May 5;13:rbag051. doi: 10.1093/rb/rbag051 (PMC13253330; doi:10.1093/rb/rbag051)
Supplement: rbag051_Supplementary_Data [file rbag051_supplementary_data.zip › Appendix A. Supplementary material.pdf]

The Sr-HA-Loaded PLGA Cage Structure Combines Cells to Construct a Bone Tissue Repair Unit

Huixing Yi [1#], Guowen Duan [1, 2#], Siyu Li [3], Dongbiao Chang [2, 3], Lulu Han [2], Yinhao Lai [2, 3], Jun Sheng [4*], Suiyan Li [1*], Jie Weng [2,3*]

1. School of Life Science and Engineering, Southwest Jiaotong University, Chengdu, Sichuan, 610031, P. R. China.
2. College of Medicine (Institute of Biomedical Engineering), Southwest Jiaotong University, Chengdu, Sichuan, 610031, P. R. China.
3. Key Laboratory of Advanced Technologies of Materials Ministry of Education, School of Materials Science and Engineering, Southwest Jiaotong University, Chengdu, Sichuan, 610031, P. R. China.
4. Department of Orthopedic, The General Hospital of Western Theater Command of PLA, Chengdu, Sichuan, 610083, P. R. China.

Correspondence: Jun Sheng, Suiyan Li, and Jie Weng.

Email: jweng@swjtu.edu.cn, lisuiyan@home.swjtu.edu.cn, and shengjunpaper@163.com

# The first two authors contributed equally to this work.

*Corresponding author.

Funding information:

This work was supported by Advanced Materials - National Science and Technology Major Project of China (2025ZD0619901), the National Key Research and Development Program of China (2023YFC2411300), and the R&D Project in Key Areas of Guangdong (No.2019B 010941002).

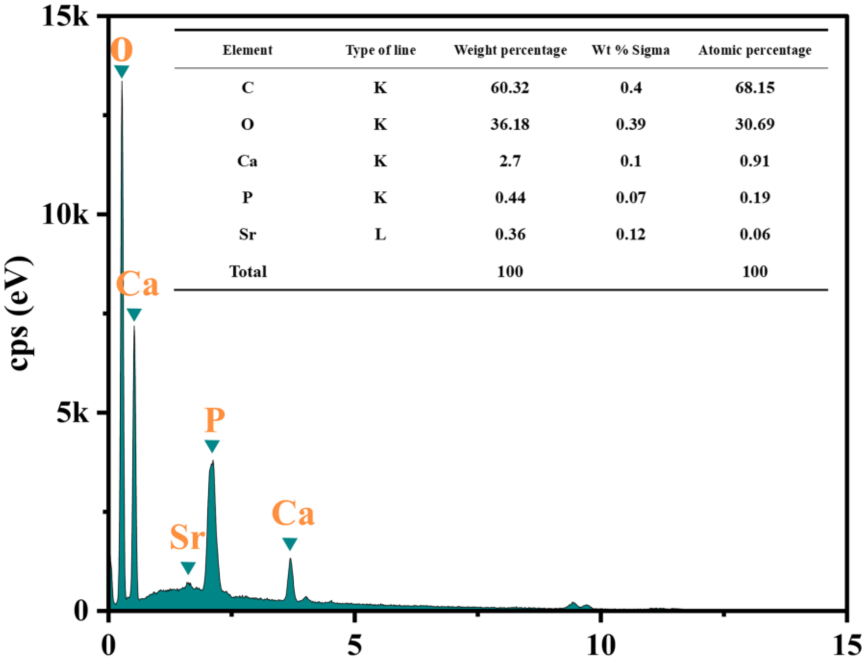

Element	Type of line	Weight percentage	Wt % Sigma	Atomic percentage
C	K	60.32	0.4	68.15
O	K	36.18	0.39	30.69
Ca	K	2.7	0.1	0.91
P	K	0.44	0.07	0.19
Sr	L	0.36	0.12	0.06
Total		100		100

Figure S1. EDS spectrum of SrHP and element distribution table of EDS.

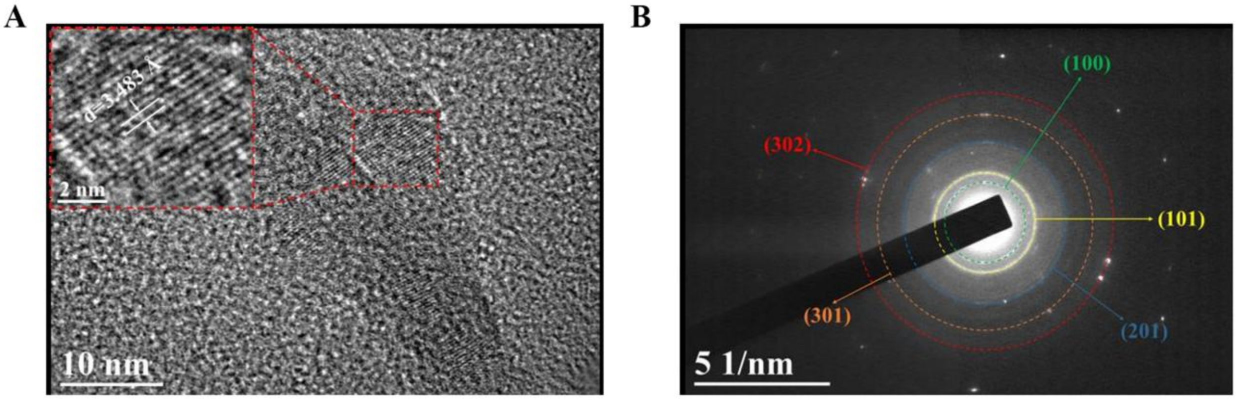

Figure S2. High-resolution TEM image and diffraction rings of Sr-HA.

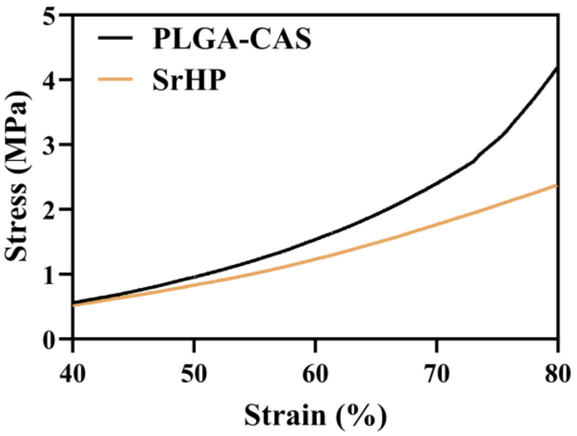

Figure S3. Stress-strain curves of PLGA-CAS and SrHP.

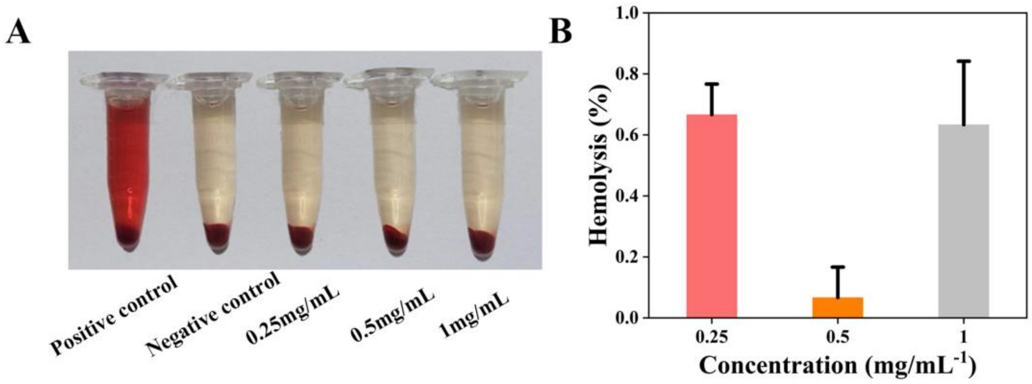

Figure S4. (A) Hemolysis test results with positive control, negative control, and SrHP at concentrations of 0.25, 0.5, and 1 mg/mL.; (B) Quantitative analysis of hemolysis rate.

Table. S1. Primer sequences for real-time fluorescence quantitative PCR of osteogenesis-related genes.

Gene	Orientation	Primer sequences
GADPH	Forward	5’- TGGAAAGCTGTGGCGTGATG -3’
	Reverse	5’- GTCAGATCCACGACGGACAC -3’
BMP-2	Forward	5’- AAGCGTCAAGCCAAACACAAACAG -3’
	Reverse	5’- GCAGCCCTCCACAACCATGTC -3’
ALP	Forward	5’- CCGGCTGGAGATGGACAAAT -3’
	Reverse	5’- TAGTCACAATGCCCACGGAC -3’
COL-I	Forward	5’- AGCACGTCTGGTTTGGAGAG-3’
	Reverse	5’- GCTGTAGGTGAAGCGACTGT-3’
OCN(BGP)	Forward	5'- CTGCGCTCTGTCTCTCTGAC -3'
	Reverse	5'- AGGGTTAAGCTCACACTGCT -3'

Table. S2. Primer sequences for real-time fluorescence quantitative PCR of angiogenesis-related genes.

Gene	Orientation	Primer sequences
GADPH	Forward	5’- ATGATTCCACCCATGGCAAATTC -3’
	Reverse	5’- TGGTTCACACCCATGACGAA -3’
VEGFα	Forward	5’- CCACACCATCACCATCGACA -3’
	Reverse	5’- CCCTCCCAACTCAAGTCCAC -3’
ANG-1	Forward	5’- TGCCATTACCAGTCAGAGGC -3’
	Reverse	5’- AGCACCGTGTAAGATCAGGC -3’

Table. S3. Primer sequences for real-time fluorescence quantitative PCR of osteoclast-related genes.

Gene	Orientation	Primer sequences
GAPDH	Forward	5’- TGGAAAGCTGTGGCGTGATG -3’
	Reverse	5’- GTCAGATCCACGACGGACAC -3’
TRAP	Forward	5’-GCGACCATTGTTAGCCACATACG-3’
	Reverse	5’-CGTTGATGTCGCACAGAGGGAT-3’
CTSK	Forward	5’-GAAGAAGACTCACCAGAAGCAG-3’
	Reverse	5’-TCCAGGTTATGGGCAGAGATT-3’
MMP9	Forward	5’-CTGGACAGCCAGACACTAAAG-3’
	Reverse	5’-CTCGCGGCAAGTCTTCAGAG-3’
